# Excess Mortality Associated with Influenza Epidemics in Portugal, 1980 to 2004

**DOI:** 10.1371/journal.pone.0020661

**Published:** 2011-06-21

**Authors:** Baltazar Nunes, Cecile Viboud, Ausenda Machado, Corinne Ringholz, Helena Rebelo-de-Andrade, Paulo Nogueira, Mark Miller

**Affiliations:** 1 Departamento de Epidemiologia, Instituto Nacional de Saúde Dr Ricardo Jorge, Lisbon, Portugal; 2 Fogarty International Center, National Institutes of Health, Bethesda, Maryland, United States of America; 3 Departamento de Doenças Infecciosas, Instituto Nacional de Saúde Dr Ricardo Jorge, Lisbon, Portugal; 4 Faculdade de Farmácia, Universidade de Lisboa, Lisbon, Portugal; University of Hong Kong, Hong Kong

## Abstract

**Background:**

Influenza epidemics have a substantial impact on human health, by increasing the mortality from pneumonia and influenza, respiratory and circulatory diseases, and all causes. This paper provides estimates of excess mortality rates associated with influenza virus circulation for 7 causes of death and 8 age groups in Portugal during the period of 1980–2004.

**Methodology/Principal Findings:**

We compiled monthly mortality time series data by age for all-cause mortality, cerebrovascular diseases, ischemic heart diseases, diseases of the respiratory system, chronic respiratory diseases, pneumonia and influenza. We also used a control outcome, deaths from injuries. Age- and cause-specific baseline mortality was modelled by the ARIMA approach; excess deaths attributable to influenza were calculated by subtracting expected deaths from observed deaths during influenza epidemic periods. Influenza was associated with a seasonal average of 24.7 all-cause excess deaths per 100,000 inhabitants, approximately 90% of which were among seniors over 65 yrs. Excess mortality was 3–6 fold higher during seasons dominated by the A(H3N2) subtype than seasons dominated by A(H1N1)/B. High excess mortality impact was also seen in children under the age of four years. Seasonal excess mortality rates from all the studied causes of death were highly correlated with each other (Pearson correlation range, 0.65 to 0.95, P<0.001) and with seasonal rates of influenza-like-illness (ILI) among seniors over 65 years (Pearson correlation rho>0.64, P<0.05). By contrast, there was no correlation with excess mortality from injuries.

**Conclusions/Significance:**

Our excess mortality approach is specific to influenza virus activity and produces influenza-related mortality rates for Portugal that are similar to those published for other countries. Our results indicate that all-cause excess mortality is a robust indicator of influenza burden in Portugal, and could be used to monitor the impact of influenza epidemics in this country. Additional studies are warranted to confirm these findings in other settings.

## Introduction

Influenza is an acute respiratory viral infection which typically occurs in the winter months in temperate areas. Seasonal influenza epidemics have a substantial mortality and morbidity impact on human health globally [1], [2], [3]. The exact burden of influenza is difficult to quantify because laboratory tests are rarely conducted on a routine basis. Further, influenza can trigger secondary bacterial infections or exacerbate existing chronic conditions, which can lead to hospitalization or death, even after the primary viral infection has been cleared. As a result, influenza disease burden studies rely on the application of statistical time series methods to broadly-defined disease outcomes, such as mortality and hospitalization from pneumonia and influenza (P&I) or respiratory and cardiovascular diseases (R&C), or all-cause mortality (ACM) [4], [5], [6], [7], [8].

Various methodological approaches have been used to determine the health burden of seasonal influenza. In general, excess mortality attributable to influenza is based on the difference between the observed mortality rate during an influenza epidemic period and a predicted baseline describing expected seasonal mortality fluctuations in the absence of influenza [9], [10]. Excess morbidity is calculated in much the same way. Statistical models, such as Poisson regression [11], multiple linear regression [12], cyclical regression [13], and autoregressive integrated moving average (ARIMA) [1], have been used to estimate excess mortality and take into account factors that are independent of influenza activity, such as seasonal variations and temporal trends in baseline mortality.

A substantial body of evidence suggests that age is one of the most important risk factors when considering the health impact of influenza. Children under 5 years and adults 65 years and older are considered to be at an increased level of risk for influenza-related complications during inter-pandemic periods [8], [14]. Several studies have explored the influenza-associated rates of hospitalization and death among seniors in the US, Canada, and Europe, as well as in a few high-income tropical settings [11], [15], [16], [17]. Very little information, however, is available for Southern Europe, with only one mortality study set in Italy [18]. In particular, no estimates for Portugal exist in the English-speaking literature. In this study, we provide estimates of excess mortality associated with influenza virus activity in Portugal by death category, age group, and circulating subtypes for 1980–2004 and compare our estimates with those from other locations.

## Materials and Methods

### Mortality and population data

Mortality data available for 1980–2004 were obtained from the Portuguese National Mortality Database of the Instituto Nacional de Estatística (National Statistics Institute in Portugal). The 9^th^ revision of the WHO International Classification of Diseases (ICD-9) was used until 2001, and the 10^th^ revision (ICD-10) was used thereafter. We compiled monthly mortality time series data according to age group (0–4, 5–54, 55–64, 65–69, 70–74, 75–79, 80–84 and ≥85 years) for all-cause mortality (ACM) and the following primary causes of death: cerebrovascular disease (ICD-9: 430–438, ICD-10: I60.0–I69.8); ischemic heart disease (ICD-9: 410–414, ICD-10: I20.0–I25.9); diseases of the respiratory system (ICD-9: 460–519, ICD-10: J00–J99); chronic respiratory diseases, including bronchitis, asthma, and emphysema (ICD-9: 490–495 and ICD-10: J41–J47); pneumonia (ICD-9: 480–486, ICD-10: J12.0–J18.9) and influenza (ICD-9: 487, ICD-10: J10.0–J11.8).

To evaluate the specificity of our approach, we also studied deaths from intentional and non-intentional injuries (ICD-9, E-codes and ICD-10, codes V-Y), which we considered as a “control” outcome with no direct causal association with influenza activity.

Age-specific annual population estimates from 1980 to 2004 were downloaded from the Instituto Nacional de Estatística website (http://www.ine.pt) [19] and used to derive monthly death rates by disease outcome and age group. Additionally, all monthly time series were standardized to a fixed number of days in the month (30.4 days). In order to cover influenza epidemic seasons, which can occur from October to May in Portugal, we defined 24 respiratory seasons starting in July of the first available year (1980) and ending in June of the last available year (2004).

### Influenza-like illness and virological surveillance data

An integrated clinical, epidemiological and virological influenza surveillance system was established in Portugal in the winter of 1990–1991. Data on influenza-like illnesses (ILI), as defined in primary care [20], were collected by a network of general Sentinel practitioners (GPs; Médicos-Sentinela) which covers approximately 2.3% of the Portuguese population. Weekly ILI incidence rates were calculated based on these data. A subset of 25–35% of GPs also provided respiratory specimens to conduct virological surveillance. Respiratory specimens were centralized at the Centro Nacional da Gripe of Instituto Nacional de Saúde Dr. Ricardo Jorge (National Influenza Centre of National Health Institute Dr. Ricardo Jorge) in Lisbon and tested for influenza A/H3N2, A/H1N1 and B subtypes by PCR or culture. Influenza subtype dominance each season was defined as the subtype that was isolated in at least 51% of the influenza-positive ILI cases.

### Definition of influenza epidemic periods

For most of the study period, 1980–81 to 2001–02, influenza epidemic periods were identified based on monthly deaths coded specifically as influenza, which is considered a specific indicator of the timing of epidemics [6], [18]. We used a Serfling cyclical regression model [13] to predict baseline mortality in the absence of influenza activity and define epidemic periods, as in [6], [18]. The model included time trends and seasonal terms and was fitted to data after exclusion of winter months (December to April), following:
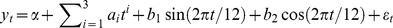
where 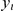 is the number of influenza-specific deaths in month t; t is a running index for time; α is the intercept; a_1_, a_2_ and a_3_ are coefficients for time trends in baseline mortality; b_1_ and b_2_ are seasonality coefficients; and 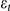 represents normally distributed errors.

Influenza epidemic periods were defined as the set of consecutive months from November to April where the observed number of influenza-specific deaths exceeds the 95% upper confidence limit of the model baseline [6], [18].

Following the transition from the 9^th^ to the 10^th^ revision of ICD in 2002, influenza-specific mortality decreased dramatically, and influenza epidemic periods could not be defined based on these data alone. For the remaining seasons 2002–2004, epidemic periods were defined by the Portuguese Influenza Surveillance System (ISS) as the weeks where ILI incidence rate exceeded the upper 95% confidence limit of the ILI baseline and where influenza virus circulation was confirmed by laboratory tests, as in [21]. Since mortality data were available on a monthly basis, we considered a given month to be epidemic if at least one week during that month was considered epidemic in the ISS. We also checked the consistency of using influenza-specific deaths and ILI incidence to define epidemic periods, based on the subset of years where both types of data were available (1991–2002).

### Estimation of influenza-associated excess deaths

A modified Serfling cyclical regression model was applied to each age group and death outcome (pneumonia, chronic respiratory diseases, all respiratory diseases, cerebrovascular diseases, ischemic heart diseases) to estimate baseline deaths in the absence of influenza activity. Influenza-associated deaths were defined as those deaths occurring in excess of the baseline predicted by a seasonal model during influenza epidemic periods [13], as explained briefly below and detailed in Text S1.

We used the Autoregressive Integrated Moving Average (ARIMA) framework to fit baseline models of the expected level of mortality in the absence of influenza virus activity and handle auto-correlation, similarly to previous efforts [22], [23] [9]. The assumption of independence of observations is rarely fulfilled by the typical cyclical linear regression approach [13].

We first replaced observed values occurring during previously-defined influenza epidemic periods by missing values, for each age group and outcome. Since ARIMA models cannot handle missing values, we replaced the missing values by the predicted values of a cyclical linear regression model (similar to [22], [24]). The cyclical regression model was fitted to the log-death rates, including harmonics and time trends up to the 5^th^ degree polynomial.

In the next step, we fitted a Seasonal-ARIMA (SARIMA) model to the augmented time series. The terms included in the SARIMA model were selected by applying an automatic algorithm using the minimal Akaike Information Criterion (AIC) [25]. The selected SARIMA model was then used to predict baseline mortality for each age group and mortality outcome.

In order to evaluate model adequacy, we computed the Box-Ljung test for autocorrelation and the Kolmogorov-Smirnov test for normality of model residuals outside of epidemic periods.

Within each epidemic period, the months with excess influenza-associated deaths were defined as those where the observed rates were above the 95% upper confidence limit of the baseline. Influenza-associated excess death rates for each of these months were estimated by taking the difference between observed and baseline rates. Finally, the cumulative number of influenza excess deaths by season was calculated as the sum of monthly influenza-associated excess deaths, after adjusting these rates for population size and true month length. As a sensitivity analysis, we repeated our estimations of excess mortality using a simple cyclical regression model, as in [6], [18].

Confidence intervals for seasonal and age-standardized excess death estimates were obtained by taking into account the uncertainty of the baseline, assuming that the sum of monthly excess deaths during epidemic periods followed a Log-normal distribution, and using the Fenton-Wilkinson method [27] (see Text S1).

Results are presented as crude and age-standardized rates using the 2000 world population as a reference [26]. We also present the proportion of deaths attributable to influenza among all deaths occurring from October to May (influenza season), averaged across all influenza seasons (IS%).

The association between seasonal age-standardized excess mortality rates from various causes, and between excess mortality and seasonal ILI attack rates (cumulative ILI rates during week 40 to week 20 of the following calendar year) was evaluated using the Pearson correlation coefficient.

All results were computed in the R Environment for Statistical Computing [28].

## Results

### Overall burden of influenza

**Figure 1:**
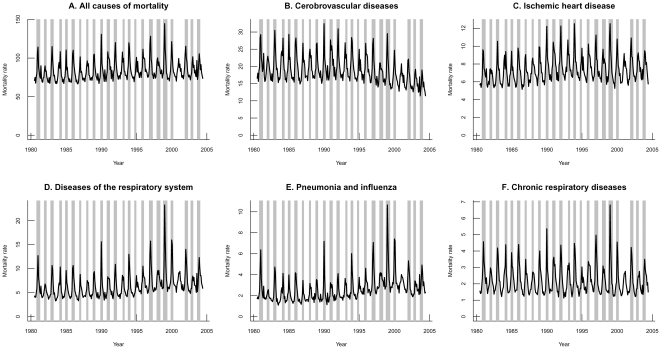
All age mortality rates for all causes, diseases of the respiratory system and pneumonia and influenza from 1980/81 to 2003/2004 in Portugal. Grey highlights represent influenza epidemic periods.

Portugal experiences highly seasonal influenza activity concentrated in winter months, with peaks in influenza-specific mortality occurring between December and March. As in other developed countries, the impact of influenza epidemics varies greatly between years, as illustrated by important year-to-year variation in the size of pneumonia and influenza and all-cause mortality peaks (Figure 1).

During the study period, 1980–2004, the seasonal average number of all cause excess deaths associated with influenza epidemics was 2,475 in Portugal (range. = 0 to 8514), 90% of which occurred in people aged ≥65 years, representing a crude excess all-cause death rate of 26 per 100,000. The corresponding average age-standardized rate was 13 per 100,000 inhabitants, representing an average of 3% of total deaths occurring between October and May (range 0 to 9.2%, Table 1). In seniors 65 years and over, the average age-standardized excess death rate during these months was 156 per 100,000 inhabitants, representing 4% of all October-May deaths in this age group (range 0 to 11.9%).

**Table 1:** Characterization of influenza seasons from 1980–1981 to 2003–2004 according to the duration of the epidemic periods, dominant (sub)type of influenza virus, influenza vaccine coverage, and influenza-like-illness (ILI) consultation attack rates in seniors over 65 years.

	Epidemic periods based on the Portuguese Influenza Surveillance System*	Epidemic periods based on influenza-specific mortality	Number of epidemic months based on influenza-specific mortality	Dominant influenza virus (sub)type**	All causes influenza-associated deaths	95% CI	All causes influenza-associated deaths rates per 100,000 population	95% CI	ILI rate per 100,000 population (65+ yrs)	Vaccine coverage (65+ yrs)
1980–1981	-	12-3	4	A(H3N2)	5638	5044–6232	39.1	34.7–43.6	-	-
1981–1982	-	1-2	2	B	0	-	0.0	-	-	-
1982–1983	-	12-2	3	A(H3N2)	5058	4477–5639	33.8	29.7–37.9	-	-
1983–1984	-	3-4	1	A(H1N1)	2487	2053–2901	15.7	12.7–18.8	-	-
1984–1985	-	1-2	2	A(H3N2)	1802	1468–2136	12.0	9.7–14.4	-	-
1985–1986	-	12-2	3	A(H3N2)	4784	4193–5375	28.4	24.6–32.1	-	-
1986–1987	-	1	1	A(H1N1)	1202	861–1543	6.7	4.7–8.7	-	-
1987–1988	-	1	1	B, A(H1N1)	0	-	0.0	-	-	-
1988–1989	-	12-1	2	A(H1N1), A(H3N2)	2530	2053–3007	13.7	11.0–16.4	-	-
1989–1990	-	1-2	2	A(H3N2)	3920	3516–4324	19.7	17.6–21.6	-	-
1990–1991	-	12-2	3	B	2781	2313–3249	13.7	11.4–16.0	-	-
1991–1992	11-1	12-2	3	A(H3N2)	2845	2466–3244	14.1	12.1–16.1	1359	-
1992–1993	2-4	3	1	B	107	17–197	0.6	0.1–1.2	1077	-
1993–1994	11-1	12-1	2	A(H3N2)	3529	3601–3997	15.9	13.8–18.0	1245	-
1994–1995	1-2	11	1	B	0	-	0.0	-	941	-
1995–1996	10-1	11-12	2	A(H3N2)	1892	1527–2257	8.8	7.0–10.7	825	-
1996–1997	11-2	12-3	4	A(H3N2)	5533	4997–6069	25.5	22.8–28.3	1094	-
1997–1998	x	3-4	2	A(H3N2)	308	91–525	1.1	0.3–1.9	1049	-
1998–1999	12-2	12-4	5	A(H3N2)	8514	7908–9120	36.1	33.2–39.0	1830	31.3
1999–2000	1-2	1-2	2	A(H3N2)	3363	2904–3822	14.2	12.1–16.2	1176	39.0
2000–2001	x	x	0	B	0	-	0.0	-	583	-
2001–2002	1-2	1-3	3	A(H3N2)	2145	1722–2568	8.8	6.9–10.8	1380	41.9
2002–2003	11-12	11-12*	2	B	0		0.0	-	677	36.9
2003–2004	10-12	10-12*	2	A(H3N2)	950	656–1244	3.1	2.1–4.1	1065	46.9

*Information is based on ILI surveillance and influenza virus activity; x – no epidemic period detected; NA, data not available.; Month numbers 1 = January to 12 = December.

**Information on the season dominant type of virus for seasons 1982–83 to 1989–90 was obtained from the World Health Organization. From 1990–91 to 2004–05 this information was obtained by the Portuguese Influenza Surveillance System.

For cerebrovascular and ischemic heart disease outcomes, the average age-standardized death rate were 2.9 and 0.7 per 100,000, respectively, representing averages of 3.2% and 2% of the age-standardized mortality rate for those causes during the influenza season (Table 2). The age-standardized mortality rates was 3.1/100,000 for respiratory diseases, 1.5/100,000 for pneumonia and influenza (P&I), and 0.8/100,000 for chronic respiratory diseases (CRD). Influenza was responsible for 9.9%, 9.3% and 6.9% on average of all deaths from P&I causes, respiratory diseases, and chronic respiratory diseases, respectively. Similar results were observed for the elderly (≥65 yrs) (Table 2). We estimate than on average 662 respiratory deaths are attributable to influenza every season in Portugal, of which 44% are coded as P&I.

**Table 2:** Average rates of excess mortality associated with influenza epidemics and proportion of deaths attributable to influenza by disease outcome, age group, and dominant viral subtype, Portugal 1980–2004.

	All causes		Cerebrovascular diseases		Ischemic heart disease		Diseases of the respiratory system		Pneumonia and Influenza		Chronic respiratory diseases	
	Rate	IS%	Rate	IS%	Rate	IS%	Rate	IS%	Rate	IS%	Rate	IS%
All individuals*	12.97	3.0	2.88	3.2	0.69	2.0	3.14	9.3	1.45	9.9	0.77	6.9
Individuals 65+*	155.75	4.0	38.48	3.5	8.19	2.3	35.69	10.1	16.86	11.6	9.09	10.0
Age-groups												
0–4	2.57	1.3	***	***	***	***	0.40	2.5	0.7	6.0	***	***
5–54	0.97	0.8	0.08	1.2	0.07	1.1	0.4	8.1	0.2	7.2	0.1	4.1
55–64	14.2	2.0	1.02	1.1	0.93	1.2	4.0	9.8	1.0	7.7	1.0	5.3
65–69	34.96	2.6	4.51	1.7	3.49	2.2	11.5	11.8	3.5	12.0	5.0	10.7
70–74	78.8	3.4	19.19	3.3	3.5	1.3	18.9	10.0	6.2	10.3	5.6	6.5
75–79	170.7	4.2	41.85	3.5	10.4	2.5	37.2	10.4	14.8	11.3	10.7	7.3
80–84	332.3	4.6	96.50	4.3	17.4	2.8	74.7	11.2	40.9	14.3	14.4	6.3
85+	825.8	5.5	207.10	4.8	33.5	3.2	174.2	11.8	103.5	14.1	33.6	8.9
Dominant subtype of virus*												
A(H3)	18.0	4.2	3.8	4.3	1.0	2.8	4.5	13.4	2.1	13.9	1.1	9.9
B or A(H1)	4.1	0.9	1.2	1.3	0.1	0.4	0.7	2.1	0.4	2.9	0.2	1.7
Ratio = A(H3)/B or A(H1)	4.4	4.5	3.1	3.4	6.8	7.5	6.8	6.5	5.5	4.8	5.0	5.7
p**	0.001	0.001	0.015	0.004	0.001	0.001	<0.001	<0.001	<0.001	<0.001	0.001	<0.001

*age-standardized rates; % IS: proportion of winter death attributable to influenza; calculated as the ratio of excess deaths to death occurring from October to May , by age group, mortality outcome, and season;

**Mann-Whitney test for comparison of excess mortality during A(H3) and A(H1) or B seasons;

***data not presented due to small death counts.

Rates are per 100,000 population.

### Age-specific estimates

**Figure 2:**
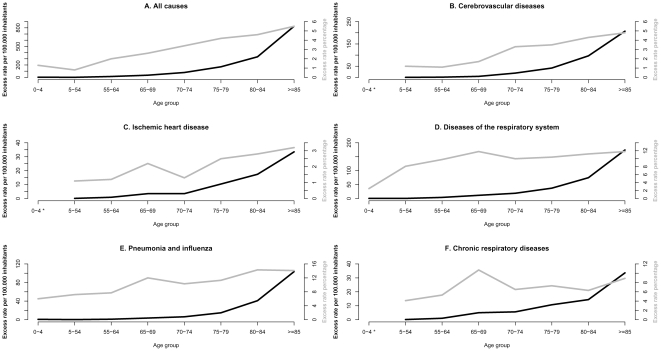
Age-specific influenza excess mortality burden. Average rates (per 100.000 persons) and proportion of winter mortality associated with influenza epidemics from 1980–1981 to 2003–2004 by age group: A. All causes, B. Cerebrovascular diseases, C. Ischemic heart diseases, D. All respiratory diseases, E. Pneumonia and Influenza (P&I), F. Chronic respiratory diseases. (* data not presented due to low annual number of deaths). The proportion of winter mortality attributable to influenza was calculated as the ratio of seasonal excess mortality to mortality occurring during Oct–Mar, for each disease outcome and age group.

The distribution of age-specific all-cause excess mortality rates followed a J-shape, with highest rates in seniors over 65 yrs, and the 0–4 age group experiencing higher excess mortality rates than individuals aged 5–54 years (Table 2 and Figure 2). In children under 5 yrs, no excess deaths could be attributed to influenza in chronic respiratory diseases, ischemic heart diseases or cerebrovascular diseases. There were very few deaths coded as such in this age group.

Excess mortality rates increased exponentially with age for age groups over 55 years. This pattern was also observed for excess mortality from P&I and CRD but not for the other causes of death studied. Interestingly, the J-shape almost disappeared when exploring the age-specific proportion of October-May deaths attributable to influenza, which instead demonstrated a near linear association with age (Figure 2). This suggests that measuring the proportion of excess deaths due to influenza is a good way to standardize burden estimates across age groups (and possibly across time and geography).

### Burden of influenza according to season and dominant sub-type

Of the 24 influenza seasons studied, 14 were dominated by the more severe A/H3N2 subtype (Table 1). Average excess mortality rates were 3.3–6.1 higher for seasons dominated by A(H3) viruses compared to seasons dominated by influenza B or A(H1), depending on the outcome studied (Table 2). Seasons associated with the highest excess mortality rates (eg, 1998–99 and 1980–81) had an especially high disease burden in people aged ≥65 years. Five of the 24 seasons were associated with no excess death in any of the studied causes.

### Comparison of influenza-related excess mortality and morbidity

**Figure 3:**
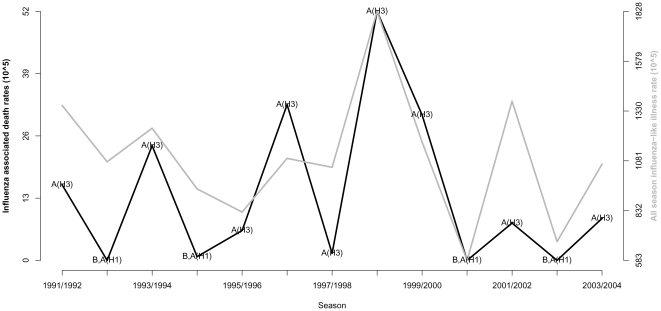
Association between seasonal rates of excess pneumonia and influenza (P&I) and seasonal rates of influenza like illnesses in the elderly population over 65 yrs.

We compared seasonal excess mortality patterns in seniors over 65 years (who account for the majority of influenza-related deaths) with morbidity patterns in ILI consultation rates in the same age group. The correlation between age-standardized influenza-associated mortality rates for the studied causes and seasonal ILI consultation rates ranged between 0.64 and 0.83 (P<0.05; Table S3). These results show that high ILI seasons were associated high excess mortality seasons among seniors. We also conducted a sensitivity analysis of the ILI data by estimating excess ILI rates occurring during epidemic periods, rather than cumulative ILI rates during Oct-May, and found very similar correlation patterns with excess mortality (Figure S1 and Tables S1 and S2). In Figure 3 we present the results for Pneumonia and Influenza.

### Influenza epidemic periods validation and model diagnostics

We used two approaches to define epidemic periods; one was based on influenza-specific deaths and the other on the Portuguese Influenza Surveillance System, combining ILI and influenza laboratory surveillance. The two approaches proved to be consistent in the period when both datasets overlapped, 1991–2002 (Table 1). There was a lag of 0 to 1 month between epidemic periods defined by the Surveillance System and influenza-specific mortality, except for the mild 1994–1995 season, where epidemic periods did not match.

Model fit was relatively good as there was no auto-correlation in the residuals of the SARIMA models in 46 out of the 48 models; however, normality of the residuals was always rejected. (See also Table S3 and figures S2–S9 for observed rates and modeled baselines.) Seasonal excess mortality estimates were generally consistent across the various causes of death studied (correlation range, 0.65 to 0.95, P<0.05; Table S4). In particular the correlation between all-causes excess deaths and excess P&I deaths was very high at 0.95 (P<0.001). Finally, we compared estimates derived from the SARIMA approach with those derived from a traditional Serfling seasonal linear regression and found very consistent results, with correlations ranging between 0.5 and 0.99 (P<0.05) depending on the mortality outcome and age group considered.

### Specificity analysis

Next, we applied the same excess mortality approach to deaths from injuries, to check that the method did not spuriously attribute deaths to influenza for unrelated outcomes. The seasonality of injuries was out-of-phase with that of influenza and displayed summer peaks, especially pronounced in 5–65 yrs. On average for the 24 seasons under study, our excess mortality approach attributed only 0.66% of all injury deaths to influenza (Table S5), which is much lower than our estimates for other studied causes of death (resp, 3–9%).

In addition, seasonal age-standardized excess mortality rates from injuries were not correlated with excess rates from causes that are traditionally associated with influenza (P>0.05 for correlation with excess deaths from P&I, chronic respiratory diseases; all respiratory diseases; cardiovascular disease; ischemic heart disease; all-causes; Table S4). Finally, there was no correlation between seasonal excess deaths from injuries and seasonal ILI attack rates in seniors.

## Discussion

This is the first study to provide estimates of influenza seasonal mortality burden in the Portuguese population, an important step in designing rational national public health measures and in comparing rates with other countries. On average, 2,475 excess all-cause deaths were attributable to influenza in Portugal each winter during 1980–2004 (range = 0 to 8514), corresponding to a seasonal excess mortality rate of 24.7 per 100,000, and a 2000 world population age-adjusted rate of 13 per 100,000.

Our estimates of influenza-related excess mortality rate are in accordance with previous studies. In the US, Thompson et al. estimated an average excess mortality rate of 19.6 per 100,000 inhabitants [8], while Schanzer et al. [16] calculated that influenza was responsible for an excess mortality rate of 13.0 per 100,000 in Canada. In Europe, excess mortality rates varied between 16.0 per 100,000 in Germany [29] and 26.0 per 100,000 in the Czech Republic [7].

Despite the fact that P&I and all-cause excess mortality are considered reliable indicators of influenza mortality impact [8], [30], here we used a more comprehensive approach and evaluated the impact of influenza epidemics on various causes of mortality in Portugal [1]. Although excess mortality was observed for 7 causes of death that are traditionally linked to influenza in this study, the highest proportion of October-May deaths attributable to influenza was observed for respiratory-related illnesses, with proportions ranging between 6.9 and 9.9%. Since influenza can precipitate other severe respiratory conditions, a higher influenza attributable proportion is expected for respiratory diseases, especially for pneumonia [31]. Similar results were obtained in Canada [16] and the US [6], [8], where pneumonia had the highest estimated percentage (8%) of deaths attributable to influenza. By contrast, all-cause mortality is a less specific outcome and we estimate that only 3% of total October-May winter deaths in Portugal can be attributable to influenza (4% in seniors over 65 years) This compares with estimates of 5% in the US [6] and 4.8% in Italy [18]. We note there is substantial inter-annual variation in this percentage in Portugal and in other countries [6], [18], as the burden of influenza epidemics varies considerably depending on circulating strains and population immunity.

It is interesting to note that P&I excess deaths only captured on average 11.2% of the total mortality burden of influenza in Portugal. By contrast, excess cerebrovascular mortality was the major contributor to total influenza-associated deaths (22.3%). This is not entirely unexpected, as cerebrovascular disease is the primary cause of death in Portugal [32]. For comparison, estimates obtained in Canada show that the proportion of excess deaths captured by P&I was 22.7% (8% for influenza and 14.7% for pneumonia), while cerebrovascular disease only contributed to 6.5% of influenza-attributable deaths [16]. The major contributor to influenza excess mortality in Canada was P&I and ischemic heart disease, the latter outcome representing 22.9% of all influenza-associated deaths. This finding is particularly relevant because ischemic heart disease is the leading cause of death in Canada [33]. Whether between-country differences in leading causes of deaths and their association with influenza result from differences in coding practices or health status remains unclear and warrants further studies.

Overall, the selection of the most appropriate cause of death to adequately measure the burden of influenza remains a point of debate. On the one hand, the usage of influenza-specific deaths grossly underestimates the actual impact of the influenza virus [34]. For example, in this study, the ratio of all influenza-attributable deaths to deaths coded specifically as influenza reached 23, and the number of influenza-coded deaths declined dramatically after 2002, in parallel with the transition of WHO International Classification of Diseases from revision 9 to 10. On the other hand, all-cause mortality lacks specificity and provides less accurate estimates, especially for milder seasons [8]. As reviewed in [1] and [35], the causal relationship between influenza and cardiovascular disease is well established, so that mortality from cardiovascular diseases should be more systematically included in influenza mortality studies.

Seasons with highest estimates of excess deaths were characterized by a dominance of the A(H3) influenza viral subtype in Portugal, a pattern that was consistent across all mortality outcomes. A higher level of hospitalizations and deaths associated with the A(H3) subtype is already well documented [6], [8], [29]. In our study, influenza B seasons had generally very low impact. In particular, the 5 seasons associated with no excess mortality were dominated by influenza B or had high proportion of B viruses. Influenza B is generally more prevalent in children and young adults, who have lower risk of severe influenza-related outcomes than seniors.

The age-standardized ratio of A(H3) vs. (A(H1) or B) excess mortality was 4.8 and 3.6 for P&I and all causes in Portuguese seniors, respectively. Similar results have been obtained in Italy (ratios of 4.5 and 2.9, respectively) [18]. By contrast, the subtype rate ratio for all-cause mortality was a much lower 2.8 in the US, after taking into account population age differences [6]. These differences could be explained by differences in the strength and sampling protocols of laboratory virus surveillance systems in each country and/or the definition of subtype-dominant seasons.

On average, 90% of influenza-associated excess deaths occurred in the ≥65 years age group. Studies in the Netherlands [36] and the US [8] estimated that 90–95% of influenza-related deaths occurred in seniors despite the use of a different methodological approach and time periods. In Portugal, the unadjusted rate of all-cause excess mortality was 165 per 100,000 on average in people ≥65 yrs. This value was 12–65% higher than estimates for Italy [18], the Netherlands [37], the US [8], and Canada [16]. One possible explanation for these differences could be the lower level of influenza vaccine coverage in Portuguese seniors compared to these countries. From 1998/99 to 2003/04, the influenza vaccine coverage in the elderly increased from 31% to 47% in Portugal [38]. While this progress is significant, these values were considerably lower than coverage estimates in the other countries over comparable time periods. For instance, seasonal influenza vaccine coverage increased from 32% in 1993/94 to 61% in 2000/2001 in Italy [18] and from 31% in 1988/89 to 65% in 2000/2001 in the US [6]. We note that no decline in influenza attributable excess mortality was observed in Italy and the US as vaccination coverage increased [6], [18], suggesting that vaccination is not the only explanation for the higher excess mortality rates observed among Portuguese seniors.

The average excess mortality rate for the children aged 0–4 years old in this study was 2.6/100,000, which is an order of magnitude higher than findings from previous studies. A recent study in the Netherlands did not find any excess mortality associated with influenza in the younger groups during 1997–2003 [37]. Another study in the US showed that the average influenza-associated excess death rates for children <1 years and 1–4 years of age were 0.3 and 0.2 per 100,000, respectively [8]. The US study also estimated a much higher rate of excess mortality due to respiratory syncytial virus (RSV) in these age groups, a finding that is in line with many other studies that establish RSV as a predominant respiratory virus in young children [39], [40], [41]. It is possible that our estimates of influenza excess mortality for young Portuguese children could be confounded by the co-circulation of RSV. Unfortunately, no information on the circulation of RSV is currently available for Portugal.

In this study, we found that excess mortality increased exponentially with age beyond 50 years, while the proportion of October-May deaths attributable to influenza increased more linearly with age. This indicates that the increased risk of influenza-related mortality with age is driven by the increase in background risk of death and may even suggest that the etiological fraction of influenza does not increase with age as much as other potential causes.

There are several caveats worth noting in our study. First, we relied on statistical models based on seasonal linear regression and SARIMA to estimate the burden of influenza, which rely on a number of assumptions. We have compared estimates from our SARIMA approach with those from a traditional Serfling seasonal linear regression [6], [24] and found very consistent results. In addition, we report a strong association between seasonal rates of influenza like illness and excess mortality for causes that are traditionally linked to influenza in the elderly population. This result confirms the robustness of our estimates of influenza-attributable excess death, since independent indicators of influenza-related excess mortality and morbidity coincide.

We have also checked the specificity of our approach by estimating excess deaths attributable to influenza in injury mortality time series. This specificity analysis revealed that the proportion of injury deaths “attributable” to influenza was very low, and that season-to-season variations in excess injuries were not associated with ILI activity. This supports the conclusion that our approach provides excess mortality estimates that are specific to influenza.

In conclusion, the present study shows that influenza epidemics in Portugal had in general the same profile as that described for other temperate countries in the Northern Hemisphere. A few issues could be further explored, in particular the potentially higher mortality burden in extreme age groups (0–4 and ≥65), compared to other countries. As in other countries, population aging tends to increase the absolute burden of influenza, which has important consequences for disease control and public health strategies. Another important point to note is the fact that the impact of influenza epidemics in Portugal was relatively well captured in the all-cause mortality time series. This result supports the use of this indicator for influenza surveillance purposes in Portugal, in particular to give near real-time estimates of inter-pandemic and pandemic influenza mortality impact [42], [43], [44]. We note that all-cause mortality is less dependent on diagnostic and coding differences between countries, time periods, or during unusual events like pandemics, as compared with cause-specific indicators. Further studies in other settings are warranted to confirm that all-cause mortality is an appropriate indicator to compare the impact of influenza epidemics at a regional and global scale [6], [45], [46].

## Supporting Information

Text S1 **Description of the method to calculate confidence limits for excess deaths and age adjusted excess deaths rates.**
(DOCX)

Figure S1 **Blue line observed weekly ILI rates, black line ILI baseline, red line upper 95% confidence limit of the ILI baseline, grey bars epidemic periods, yellow bars periods with excess ILI consultations attributable to influenza.**
(TIFF)

Figure S2 **Mortality rates (blue), mortality baseline (black) and 95% confidence limit (red), estimated excess death rate (green) by month and influenza epidemic periods (grey rectangles) for the study causes of death – age group 85+.**
(TIFF)

Figure S3 **Mortality rates (blue), mortality baseline (black) and 95% confidence limit (red), estimated excess death rate (green) by month and influenza epidemic periods (grey rectangles) for the study causes of death – age group 80–84.**
(TIFF)

Figure S4 **Mortality rates (blue), mortality baseline (black) and 95% confidence limit (red), estimated excess death rate (green) by month and influenza epidemic periods (grey rectangles) for the study causes of death – age group 75–79.**
(TIFF)

Figure S5 **Mortality rates (blue), mortality baseline (black) and 95% confidence limit (red), estimated excess death rate (green) by month and influenza epidemic periods (grey rectangles) for the study causes of death – age group 70–74.**
(TIFF)

Figure S6 **Mortality rates (blue), mortality baseline (black) and 95% confidence limit (red), estimated excess death rate (green) by month and influenza epidemic periods (grey rectangles) for the study causes of death – age group 65–69.**
(TIFF)

Figure S7 **Mortality rates (blue), mortality baseline (black) and 95% confidence limit (red), estimated excess death rate (green) by month and influenza epidemic periods (grey rectangles) for the study causes of death – age group 55–64.**
(TIFF)

Figure S8 **Mortality rates (blue), mortality baseline (black) and 95% confidence limit (red), estimated excess death rate (green) by month and influenza epidemic periods (grey rectangles) for the study causes of death – age group 5–54.**
(TIFF)

Figure S9 **Mortality rates (blue), mortality baseline (black) and 95% confidence limit (red), estimated excess death rate (green) by month and influenza epidemic periods (grey rectangles) for the study causes of death – age group 0–4.**
(TIFF)

Table S1 **Comparison between seasonal ILI attack rates (cumulative rates for week 40–20) and seasonal excess ILI rates occurring during epidemic periods.** Rates are per 100,000.
(DOCX)

Table S2 **Correlation matrix between seasonal age-standardized excess mortality rates, cumulative ILI attack rates (week 40 to wee 20), and excess ILI rates during the influenza epidemic period.**
(DOCX)

Table S3 **Seasonal ARIMA best-fitted models by R package forecast and Box-Ljong test for residuals auto correlation.**
(DOCX)

Table S4 **Correlation matrix between seasonal age-standardized excess rates.** Injuries are used as a control time series which should not be associated with influenza virus circulation.
(DOCX)

Table S5 **Sensitivity analysis: excess deaths and age-standardized excess death rates from injuries that are “attributable to influenza” by our method.** Injuries comprise all external causes of death.
(DOCX)
