# Supplementary material for: Excess Mortality Associated with Influenza Epidemics in Portugal, 1980 to 2004
Source: PLoS One. 2011 Jun 21;6(6):e20661. doi: 10.1371/journal.pone.0020661 (PMC3119666; doi:10.1371/journal.pone.0020661)
Supplement: Text S1 — Description of the method to calculate confidence limits for excess deaths and age adjusted excess deaths rates. (DOCX) [file pone.0020661.s001.docx]

Supplementary text S1

**Description of the method to calculate confidence limits for excess deaths and age adjusted excess deaths rates**

Let consider:

- $y_{tag}$ the number of observed deaths in month t (1…12) of flu-year a (1…24) and age group g (1…8);
- $E_{a}$ the estimate of the epidemic period of flu-year a
- $D_{ag}$ the periods of months where an excess of deaths in $y_{tag}$ is attributed to the epidemic period of flu-year a

1. Compute the monthly rate of deaths adjusted for a 30.4 days month:

$$r_{tag}=\left( \frac{y_{ta}}{m_{t}}30.4 \right)/{N_{ag}}$$

2. Compute the time series $z_{tag}=ln\left( r_{tag} \right)$ in order to stabilize de variance;
3. Compute $z_{tag}^{*}$the time series of natural logarithm of the death rare in month t (1…12) of flu-year a (1…24) and age group g (1…8) without the epidemic periods $E_{a}$, i.e. $z_{tag}^{*}=\left\{ \left( t,a \right)\notin E_{a} \right\}$;
4. For each age group g (1…8) a cyclical regression model is fitted to $z_{tag}^{*}$, the model is then used to predict the number of deaths for the $E_{a}$ periods. A new time series $z_{tag}^{'}$is then build by inputting the missing values of $z_{tag}^{*}$ with cyclical regression model predictions;
5. For each age group g an seasonal ARIMA model is fitted to the time series $z_{tag}^{'}$. Then compute the time series $\hat{z}_{tag}^{'}$ representing the fitted values using the adjusted seasonal ARIMA model will represent the natural logarithm of the monthly death rate baseline without the effect of the epidemic periods $E_{a}$ and the respective upper 95% confidence limit given by $\hat{z}_{tag}^{'}+p_{0.975}\hat{\sigma}_{eg}$, where $\hat{\sigma}_{eg}$ is the standard deviation of the seasonal ARIMA model residuals and $p_{0.975}$ the 0.975 percentile of the standard normal distribution;
6. Log baseline and upper 95% confidence limits are anti log:

$$r_{tag}^{'}=exp\left( \hat{z}_{tag}^{'} \right)$$

$${rup}_{tag}^{'}=exp\left( \hat{z}_{tag}^{'}+p_{0.975}\hat{\sigma}_{eg} \right)$$

7. The $D_{ag}$ are then obtained as the periods included in $E_{a}$ where

$$r_{tag}>{rup}_{tag}^{'}$$

8. Compute excess rate and absolute excess deaths attributable to influenza epidemics for $\left( t,a,g \right)\in D_{ag}$:

Excess rate - ${exc\_r}_{tag}=r_{tag}-r_{tag}^{'}$

Absolute excess deaths - ${exc\_y}_{tag}={exc\_r}_{tag}\times A_{tag}$

where $A_{tag}=\frac{m_{t}\times N_{ag}}{30.4}$

1. Compute total excess deaths in the epidemic period $E_{a}$ for age group g, i.e. in period $D_{ag}$:

Absolute excess deaths in $D_{ag}$: ${exc\_y}_{ag}=\sum_{t\in D_{ag}} {exc\_y}_{tag}$

1. Compute total excess deaths in epidemic period $E_{a}$ (all age groups)

$${exc\_y}_{a}=\sum_{g=1}^{8} {exc\_y}_{ag}$$

2. Compute age-standardized excess rates for epidemic period $E_{a}$:

$${exc\_r}_{a}=\sum_{g=1}^{8} w_{g}\times\frac{{exc\_y}_{ag}}{N_{ag}}$$

Where $w_{g}$ is the weight of age group g in the reference population used (world population 2000);

1. Confidence interval for the total excess deaths in epidemic period $E_{a}$ - ${exc\_y}_{a}$:

$${exc\_y}_{a}=\sum_{g=1}^{8} {exc\_y}_{ag}$$

Let start by finding the distribution of ${exc\_y}_{ag}$, if

$${exc\_y}_{ag}=\sum_{t,a\in D_{ag}} \left( r_{tag}-r_{tag}^{'} \right)\times A_{tag}=\sum_{t,a\in D_{ag}} r_{tag}A_{tag}-\sum_{t,a\in D_{ag}} r_{tag}^{'}A_{tag}$$

Assuming that the observed rates $r_{tag}$are fixed, we only need to find the distribution of $\sum_{t,a\in D_{ag}} r_{tag}^{'}A_{tag}$.

From the seasonal ARIMA model we know that $\hat{z}_{tag}^{'}\sim N\left( z_{tag}^{'},\sigma_{eg}^{2} \right)$ so

$$r_{tag}^{'}=e^{\hat{z}_{tag}^{'}}\sim Log-N\left( z_{tag}^{'},\sigma_{eg}^{2} \right)$$

and

$${A_{tag}r}_{tag}^{'}\sim Log-N\left( {ln\left( A_{tag} \right)+z}_{tag}^{'},\sigma_{eg}^{2} \right)$$

And by the Fenton and Wilkinson approximation

$$\sum_{t,a\in D_{ag}} r_{tag}^{'}A_{tag}=\sum_{t,a\in D_{ag}} y_{tag}^{'}\sim Log-N\left( \mu_{y_{ag}^{'}},\sigma_{y_{ag}^{'}}^{2} \right)$$

Where

$$\sigma_{y_{ag}^{'}}^{2}=ln\left( \left( e^{\sigma_{eg}^{2}}-1 \right)\frac{\sum_{t,a\in D_{ag}} e^{2\left( {ln\left( A_{tag} \right)+z}_{tag}^{'} \right)}}{\left[ \sum_{t,a\in D_{ag}} e^{{ln\left( A_{tag} \right)+z}_{tag}^{'}} \right]^{2}}+1 \right)$$

and

$\mu_{y_{ag}^{'}}=ln\left( \sum_{t,a\in D_{ag}} e^{{ln\left( A_{tag} \right)+z}_{tag}^{'}} \right)+\frac{\sigma_{eg}^{2}}{2}-\frac{\sigma_{y_{ag}^{'}}^{2}}{2}$

Consider now:

$${exc\_y}_{a}=\sum_{g=1}^{8} {exc\_y}_{ag}=\sum_{g=1}^{8} \left[ \sum_{t,a\in D_{ag}} y_{tag}-\sum_{t,a\in D_{ag}} r_{tag}^{'}A_{tag} \right]=$$

$$=\sum_{g=1}^{8} \sum_{t,a\in D_{ag}} y_{tag}-\sum_{g=1}^{8} \sum_{t,a\in D_{ag}} r_{tag}^{'}A_{tag}$$

Considering that $\sum_{g=1}^{8} \sum_{t,a\in D_{ag}} y_{tag}$ is fixed then we must find the distribution of $\sum_{g=1}^{8} \sum_{t,a\in D_{ag}} r_{tag}^{'}A_{tag}=\sum_{g=1}^{8} \sum_{t,a\in D_{ag}} y_{tag}^{'}=\sum_{g=1}^{8} y_{ag}^{'}$.

From the results above we know that $y_{ag}^{'}\sim Log-N\left( \mu_{y_{ag}^{'}},\sigma_{y_{ag}^{'}}^{2} \right)$ so:

$$\sum_{g=1}^{8} y_{ag}^{'}\sim Log-N\left( \mu_{y_{a}^{'}},\sigma_{y_{a}^{'}}^{2} \right)$$

Where by the Fenton and Wilkinson approximation

$$\sigma_{y_{a}^{'}}^{2}=ln\left[ \frac{\sum_{g=1}^{8} e^{2\mu_{y_{ag}^{'}}+\sigma_{y_{ag}^{'}}^{2}}\left( e^{\sigma_{y_{ag}^{'}}^{2}}-1 \right)}{\left( \sum_{g=1}^{8} e^{\mu_{y_{ag}^{'}}+{\sigma_{y_{ag}^{'}}^{2}}/2} \right)^{2}}+1 \right]$$

$$\mu_{y_{a}^{'}}=ln\left( \sum_{g=1}^{8} e^{\mu_{y_{ag}^{'}}+{\sigma_{y_{ag}^{'}}^{2}}/2} \right)-\frac{\sigma_{y_{a}^{'}}^{2}}{2}$$

The 95%confidence limits for ${exc\_y}_{a}$ are computed using the upper 0.975 and lower 0.025 probability quantiles of the Log-N distribution with $Log-N\left( \mu_{y_{a}^{'}},\sigma_{y_{a}^{'}}^{2} \right)$

1. –Confidence interval for standardized excess rates for epidemic period $E_{a}$:

$${exc\_r}_{a}=\sum_{g=1}^{8} w_{g}\times\frac{{exc\_y}_{ag}}{N_{ag}}=\sum_{g=1}^{8} W_{ag}\times{exc\_y}_{ag}=$$

Where $W_{ag}=\frac{w_{g}}{N_{ag}}$

$$=\sum_{g=1}^{8} W_{ag}y_{ag}-\sum_{g=1}^{8} W_{ag}y_{ag}^{'}=$$

$$W_{ag}y_{ag}^{'}\sim Log-N\left( ln\left( W_{ag} \right)+\mu_{y_{ag}^{'}},\sigma_{y_{ag}^{'}}^{2} \right)$$

Where by the Fenton and Wilkinson approximation

$${exc\_r}_{a}\sim Log-N\left( {\mu_{r}}_{a},\sigma_{r_{a}}^{2} \right)$$

$$\sigma_{r_{a}}^{2}=ln\left[ \frac{\sum_{g=1}^{8} e^{2ln\left( W_{ag} \right)+\mu_{y_{ag}^{'}}+\sigma_{y_{ag}^{'}}^{2}}\left( e^{\sigma_{y_{ag}^{'}}^{2}}-1 \right)}{\left( \sum_{g=1}^{8} e^{ln\left( W_{ag} \right)+\mu_{y_{ag}^{'}}+{\sigma_{y_{ag}^{'}}^{2}}/2} \right)^{2}}+1 \right]$$

$${\mu_{r}}_{a}= ln\left( \sum_{g=1}^{8} e^{ln\left( W_{ag} \right)+\mu_{y_{ag}^{'}}+{\sigma_{y_{ag}^{'}}^{2}}/2} \right)-\frac{\sigma_{r_{a}}^{2}}{2}$$

The 95%confidence limits for ${exc\_y}_{a}$ are computed using the upper 0.975 and lower 0.025 probability quantiles of the Log-N distribution with $Log-N\left( \mu_{y_{a}^{'}},\sigma_{y_{a}^{'}}^{2} \right)$
