# Supplementary figures and images for: Excess Mortality Associated with Influenza Epidemics in Portugal, 1980 to 2004
Source: PLoS One. 2011 Jun 21;6(6):e20661. doi: 10.1371/journal.pone.0020661 (PMC3119666; doi:10.1371/journal.pone.0020661)

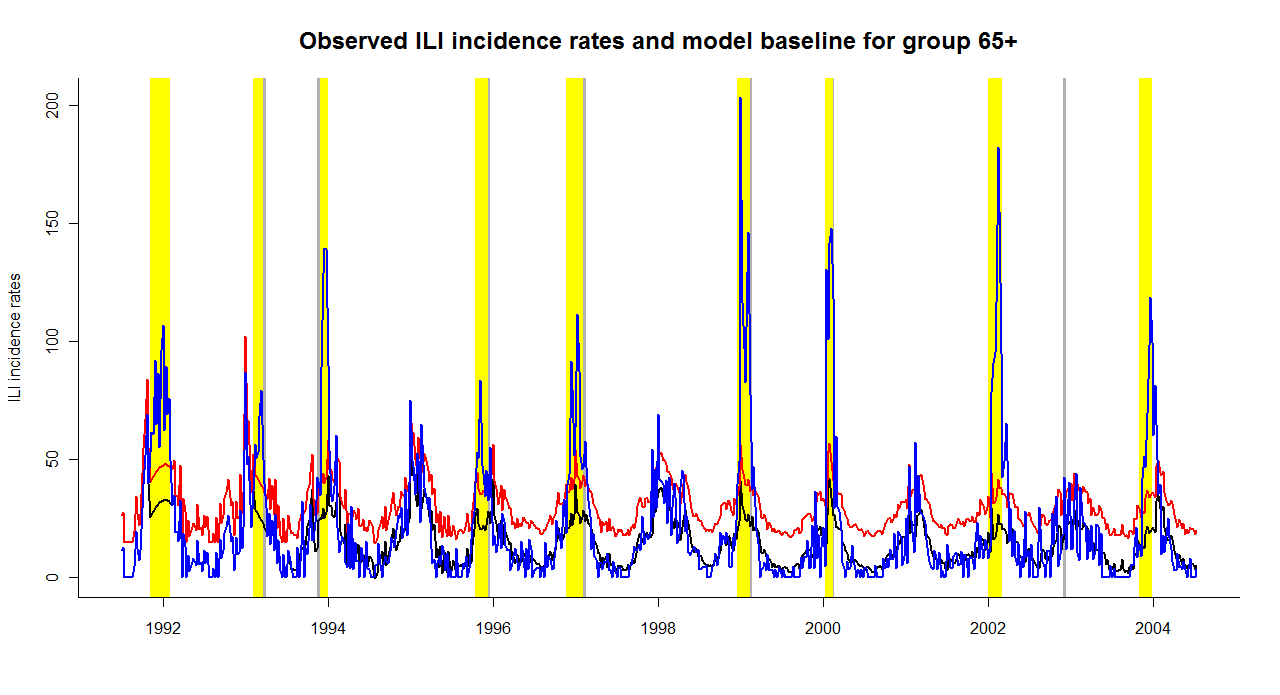

Supplement: Figure S1 — Blue line observed weekly ILI rates, black line ILI baseline, red line upper 95% confidence limit of the ILI baseline, grey bars epidemic periods, yellow bars periods with excess ILI consultations attributable to influenza. (TIFF) [file pone.0020661.s002.tiff]

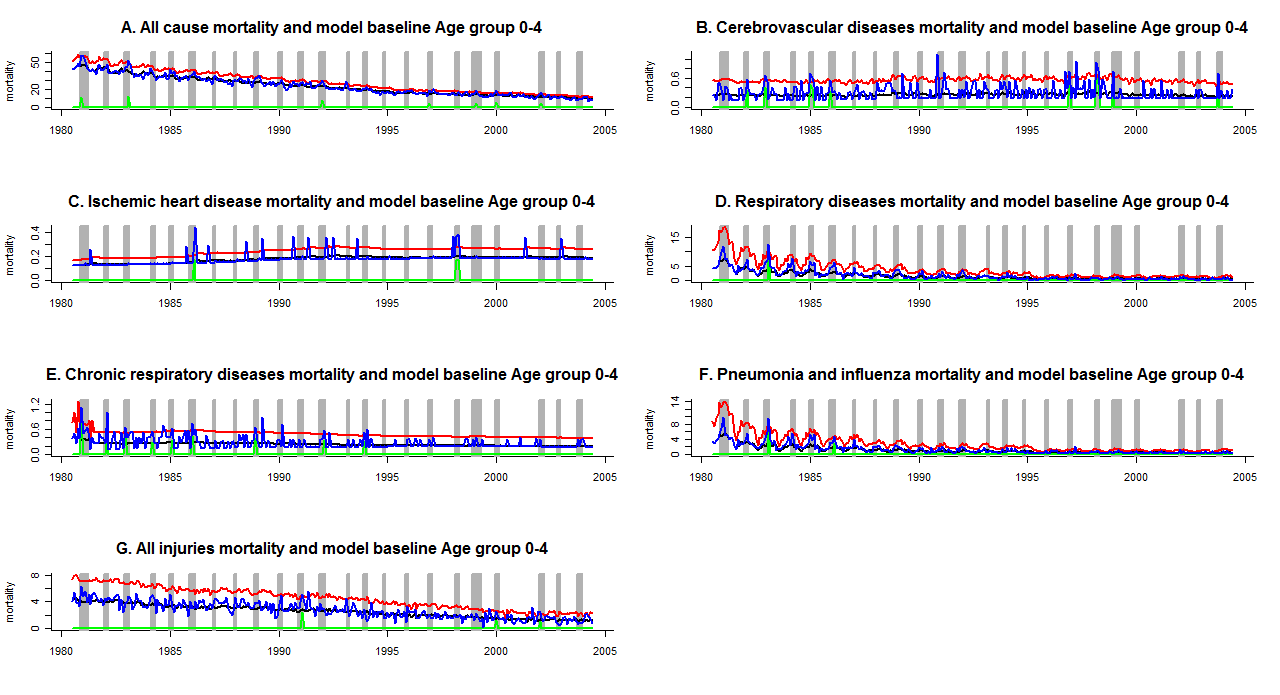

Supplement: Figure S2 — Mortality rates (blue), mortality baseline (black) and 95% confidence limit (red), estimated excess death rate (green) by month and influenza epidemic periods (grey rectangles) for the study causes of death – age group 85+. (TIFF) [file pone.0020661.s003.tiff]

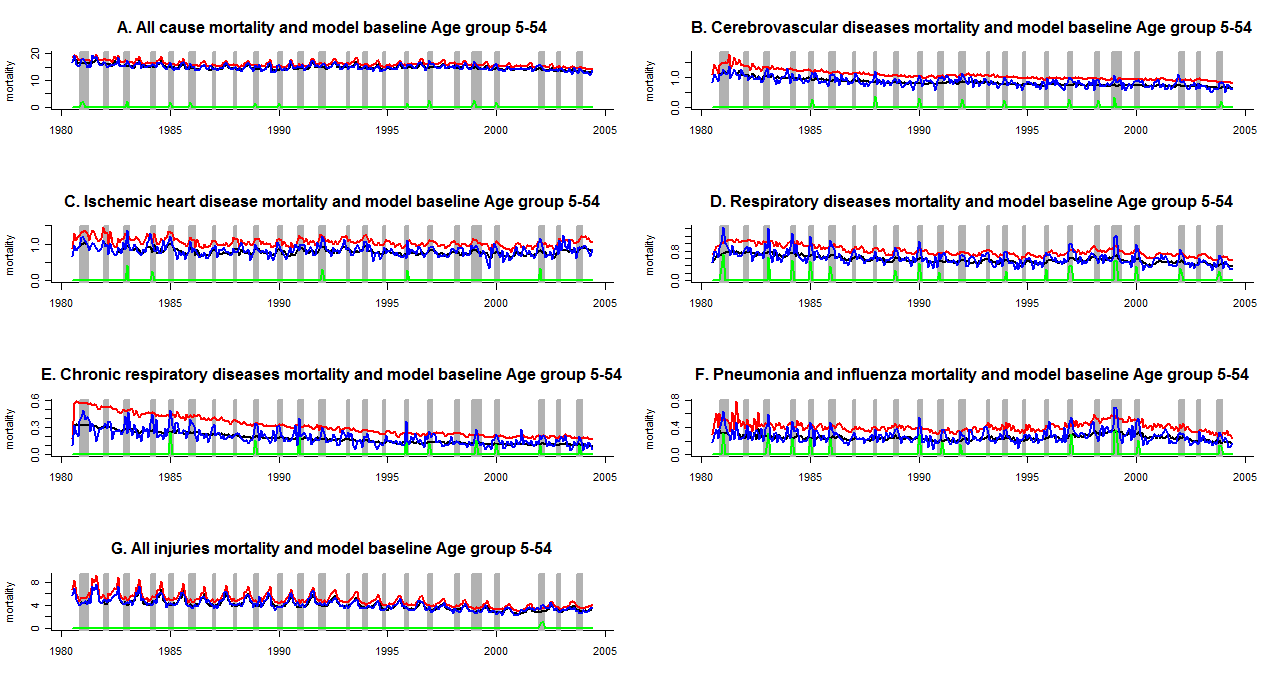

Supplement: Figure S3 — Mortality rates (blue), mortality baseline (black) and 95% confidence limit (red), estimated excess death rate (green) by month and influenza epidemic periods (grey rectangles) for the study causes of death – age group 80–84. (TIFF) [file pone.0020661.s004.tiff]

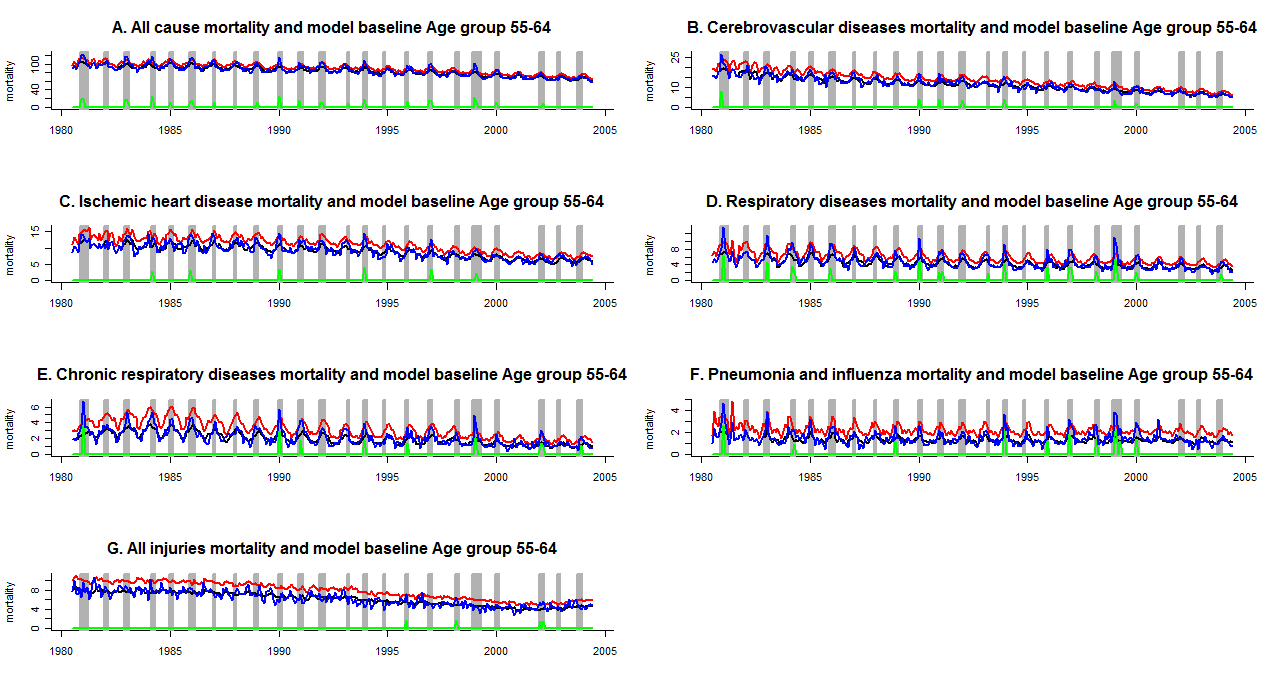

Supplement: Figure S4 — Mortality rates (blue), mortality baseline (black) and 95% confidence limit (red), estimated excess death rate (green) by month and influenza epidemic periods (grey rectangles) for the study causes of death – age group 75–79. (TIFF) [file pone.0020661.s005.tiff]

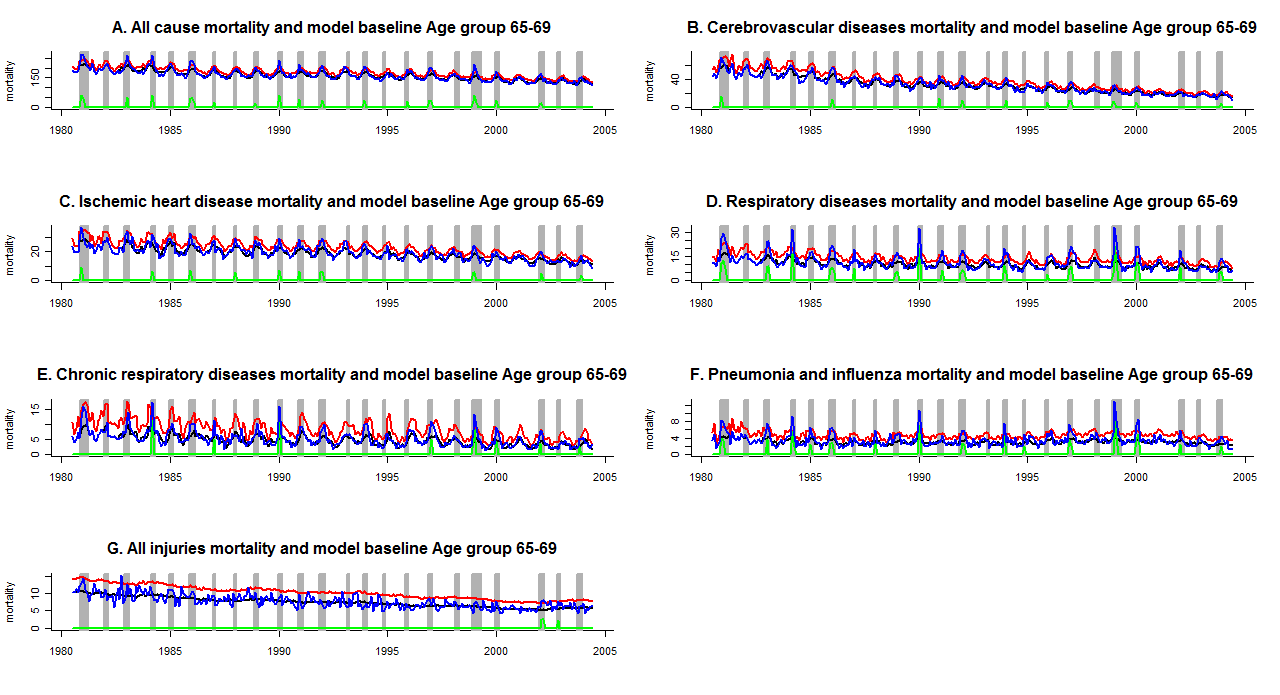

Supplement: Figure S5 — Mortality rates (blue), mortality baseline (black) and 95% confidence limit (red), estimated excess death rate (green) by month and influenza epidemic periods (grey rectangles) for the study causes of death – age group 70–74. (TIFF) [file pone.0020661.s006.tiff]

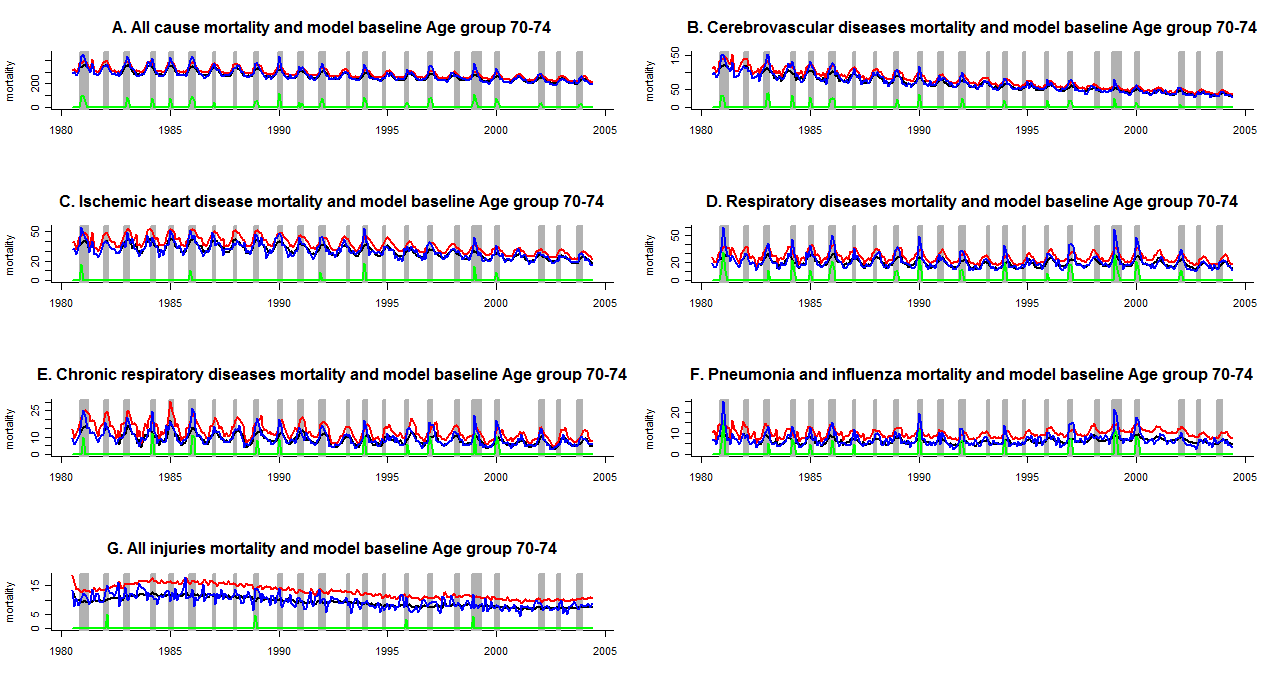

Supplement: Figure S6 — Mortality rates (blue), mortality baseline (black) and 95% confidence limit (red), estimated excess death rate (green) by month and influenza epidemic periods (grey rectangles) for the study causes of death – age group 65–69. (TIFF) [file pone.0020661.s007.tiff]

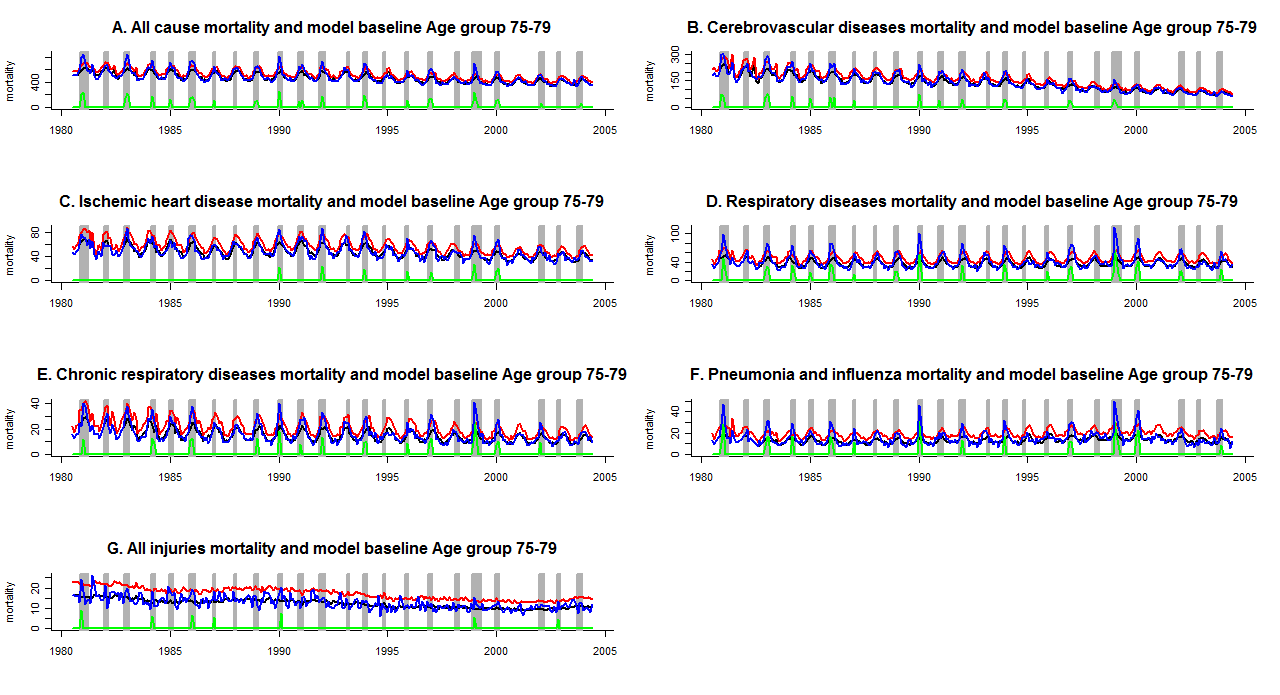

Supplement: Figure S7 — Mortality rates (blue), mortality baseline (black) and 95% confidence limit (red), estimated excess death rate (green) by month and influenza epidemic periods (grey rectangles) for the study causes of death – age group 55–64. (TIFF) [file pone.0020661.s008.tiff]

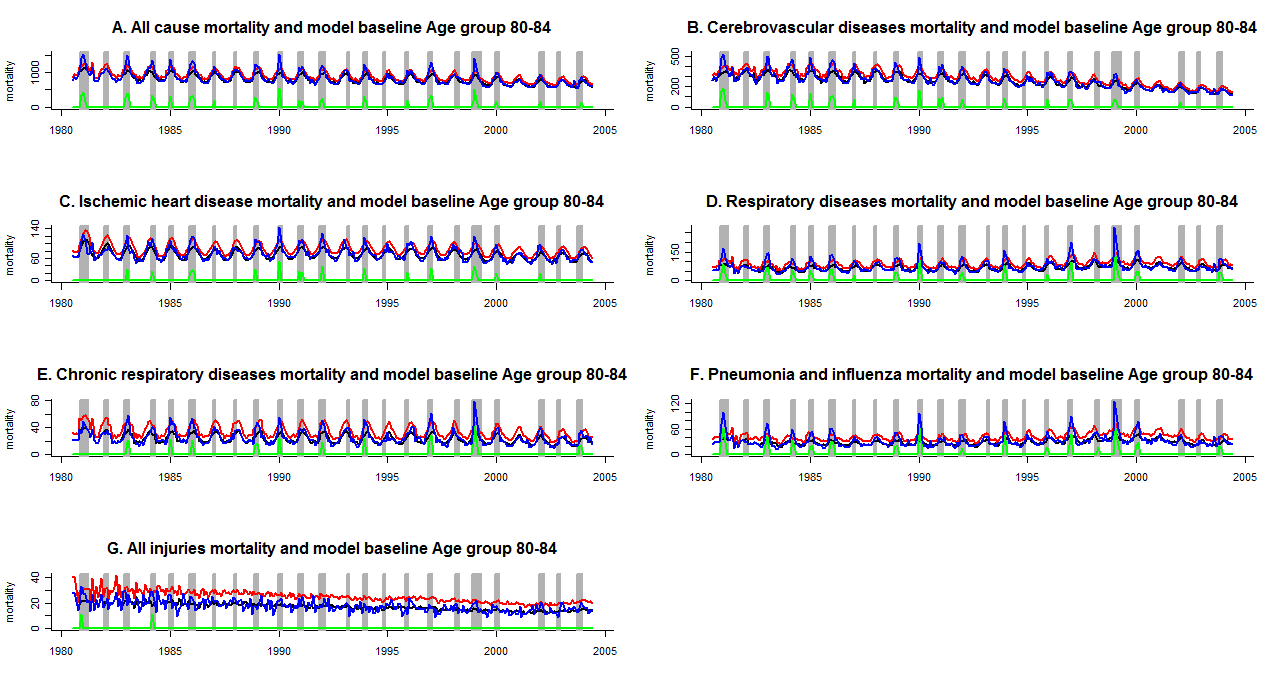

Supplement: Figure S8 — Mortality rates (blue), mortality baseline (black) and 95% confidence limit (red), estimated excess death rate (green) by month and influenza epidemic periods (grey rectangles) for the study causes of death – age group 5–54. (TIFF) [file pone.0020661.s009.tiff]

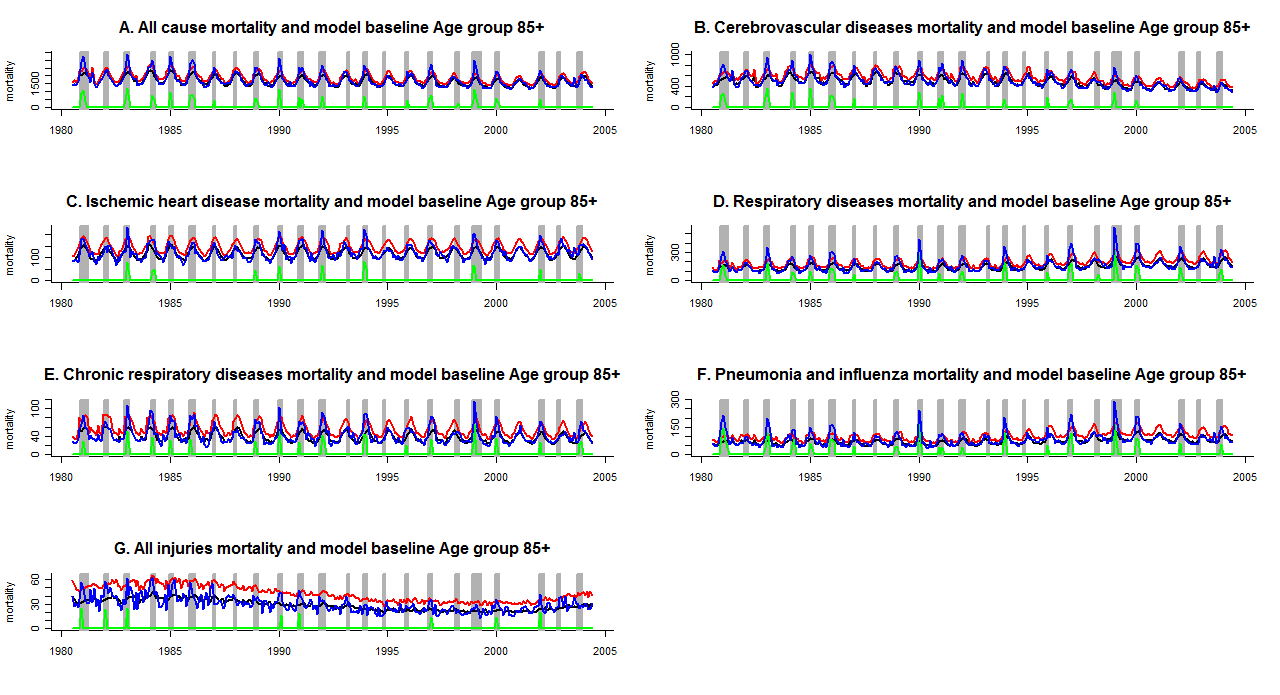

Supplement: Figure S9 — Mortality rates (blue), mortality baseline (black) and 95% confidence limit (red), estimated excess death rate (green) by month and influenza epidemic periods (grey rectangles) for the study causes of death – age group 0–4. (TIFF) [file pone.0020661.s010.tiff]
