# Supplementary material for: Excess Mortality Associated with Influenza Epidemics in Portugal, 1980 to 2004
Source: PLoS One. 2011 Jun 21;6(6):e20661. doi: 10.1371/journal.pone.0020661 (PMC3119666; doi:10.1371/journal.pone.0020661)
Supplement: Table S1 — Comparison between seasonal ILI attack rates (cumulative rates for week 40–20) and seasonal excess ILI rates occurring during epidemic periods. Rates are per 100,000. (DOCX) [file pone.0020661.s011.docx]

Table S1: Comparison between seasonal ILI attack rates (cumulative rates for week 40-20) and seasonal excess ILI rates occurring during epidemic periods. Rates are per 100,000

| **Season** | **Cumulative ILI attack rate (week 40-20)** | **Excess ILI during epidemic periods** |
| --- | --- | --- |
| 1991-92 | 1359 | 596 |
| 1992-93 | 1077 | 221 |
| 1993-94 | 1245 | 460 |
| 1994-95 | 941 | 0 |
| 1995-96 | 825 | 238 |
| 1996-97 | 1094 | 475 |
| 1997-98 | 1049 | 0 |
| 1998-99 | 1830 | 696 |
| 1999-00 | 1176 | 410 |
| 2000-01 | 583 | 0 |
| 2001-02 | 1380 | 756 |
| 2002-03 | 677 | 0 |
| 2003-04 | 1065 | 503 |
