# Supplementary material for: Excess Mortality Associated with Influenza Epidemics in Portugal, 1980 to 2004
Source: PLoS One. 2011 Jun 21;6(6):e20661. doi: 10.1371/journal.pone.0020661 (PMC3119666; doi:10.1371/journal.pone.0020661)
Supplement: Table S2 — Correlation matrix between seasonal age-standardized excess mortality rates, cumulative ILI attack rates (week 40 to wee 20), and excess ILI rates during the influenza epidemic period. (DOCX) [file pone.0020661.s012.docx]

Table S2: Correlation matrix between seasonal age-standardized excess mortality rates, cumulative ILI attack rates (week 40 to wee 20), and excess ILI rates during the influenza epidemic period.

|  | Cumulative Oct-May ILI rate | Excess ILI rate during epidemc periods |
| --- | --- | --- |
| All causes | 0.765* | 0.708* |
| CVD | 0.641* | 0.605* |
| IHD | 0.829* | 0.707* |
| DRS | 0.807* | 0.751* |
| PI | 0.743* | 0.648* |
| CRD | 0.794* | 0.722* |
| Injuries | 0.426 | 0.514 |

CVD: cardiovascular disease; IHD: ischemic heart disease; DRS: diseases of the respiratory system: PI: Pneumonia and Influenza; CRD chronic respiratory disease: * p<0.05;
