# Supplementary material for: Excess Mortality Associated with Influenza Epidemics in Portugal, 1980 to 2004
Source: PLoS One. 2011 Jun 21;6(6):e20661. doi: 10.1371/journal.pone.0020661 (PMC3119666; doi:10.1371/journal.pone.0020661)
Supplement: Table S3 — Seasonal ARIMA best-fitted models by R package forecast and Box-Ljong test for residuals auto correlation. (DOCX) [file pone.0020661.s013.docx]

Table S3 Seasonal ARIMA best-fitted models by R package forecast and Box-Ljong test for residuals auto correlation

| Cause | Model | Box-Ljung test for auto-correlation of residuals |
| --- | --- | --- |
| All causes |  |  |
| 0-4 | ARIMA(0,1,2)(1,0,1)[12] | X= 2.5754, df = 5.663, p = 0.8321 |
| 5-54 | ARIMA(2,1,1)(2,0,2)[12] | X = 3.2763, df = 5.663, p = 0.7379 |
| 55-64 | ARIMA(2,1,2)(1,0,1)[12] | X = 4.3128, df = 5.663, p = 0.5929 |
| 65-69 | ARIMA(1,1,2)(2,0,1)[12] | X = 3.4083, df = 5.663, p = 0.7194 |
| 70-74 | ARIMA(4,1,2)(2,0,1)[12] | X = 1.1144, df = 5.663, p = 0.9739 |
| 75-79 | ARIMA(2,1,1)(2,0,0)[12] | X = 6.3192, df = 5.663, p = 0.34 |
| 80-84 | ARIMA(1,1,2)(2,0,1)[12] | X = 2.9072, df = 5.663, p = 0.7886 |
| 85+ | ARIMA(1,1,1)(2,0,1)[12] | X = 6.7818, df = 5.663, p = 0.3053 |
| Diseases of respiratory system |  |  |
| 0-4 | ARIMA(1,1,2)(1,0,1)[12] | X= 0.201, df = 5.663, p= 0.9997 |
| 5-54 | ARIMA(2,1,1)(0,0,2)[12] | X = 4.3446, df = 5.663, p= 0.5885 |
| 55-64 | ARIMA(0,1,3)(0,0,2)[12] | X = 8.1654, df = 5.663, p = 0.1979 |
| 65-69 | ARIMA(1,1,1)(2,0,0)[12] | X = 4.356, df = 5.663, p = 0.5869 |
| 70-74 | ARIMA(2,1,1)(2,0,1)[12] | X = 1.5089, df = 5.663, p = 0.9465 |
| 75-79 | ARIMA(1,0,0)(2,0,2)[12] | X = 2.429, df = 5.663, p= 0.8504 |
| 80-84 | ARIMA(1,1,1)(1,0,1)[12] | X = 2.5779, df = 5.663, p = 0.8318 |
| 85+ | ARIMA(5,1,3)(2,0,1)[12] | X = 0.7527, df = 5.663, p = 0.9902 |
| Chronic respiratory diseases |  |  |
| 0-4 | ARIMA(0,1,1)(1,0,0)[12] | X = 8.7101, df = 5.663, p = 0.1654 |
| 5-54 | ARIMA(1,1,1)(0,0,2)[12] | X = 7.2876, df = 5.663, p = 0.26 |
| 55-64 | ARIMA(2,1,2)(1,0,1)[12] | X = 4.8272, df = 5.663, p = 0.5239 |
| 65-69 | ARIMA(0,1,4)(0,0,2)[12] | X = 5.4006, df = 5.663, p = 0.451 |
| 70-74 | ARIMA(1,1,0)(2,0,1)[12] | X = 29.0582, df = 5.663, p <0.001 |
| 75-79 | ARIMA(4,1,1)(2,0,1)[12] | X = 4.0111, df = 5.663, p = 0.6347 |
| 80-84 | ARIMA(2,1,1)(2,0,1)[12] | X = 2.4044, df = 5.663, p = 0.8535 |
| 85+ | ARIMA(3,0,4)(1,0,1)[12] | X = 2.917, df = 5.663, p = 0.787 |
| Pneumonia and Influenza |  |  |
| 0-4 | ARIMA(3,1,3) | X= 1.6593, df = 5.663, p = 0.9336 |
| 5-54 | ARIMA(2,0,1)(0,0,1)[12] | X = 3.4964, df = 5.663, p = 0.707 |
| 55-64 | ARIMA(2,0,1)(1,0,1)[12] | X = 6.496, df = 5.663, p = 0.332 |
| 65-69 | ARIMA(2,0,1)(2,0,2)[12] | X = 4.8612, df = 5.663, p = 0.5194 |
| 70-74 | ARIMA(3,0,3)(2,0,2)[12] | X = 1.7874, df = 5.663, p = 0.9216 |
| 75-79 | ARIMA(1,0,2)(2,0,2)[12] | X = 1.7948, df = 5.663, p = 0.9209 |
| 80-84 | ARIMA(2,1,1)(2,0,2)[12] | X = 2.6166, df = 5.663, p = 0.8268 |
| 85+ | ARIMA(0,1,1)(1,0,1)[12] | X = 1.2253, df = 5.663, p = 0.9672 |
| Cardiovascular disease |  |  |
| 0-4 | ARIMA(1,0,1)(1,0,1)[12] | X= 9.8662, df = 5.663, p= 0.1114 |
| 5-54 | ARIMA(0,1,2)(1,0,2)[12] | X = 5.4022, df = 5.663, p = 0.4516 |
| 55-64 | ARIMA(2,1,2)(1,0,1)[12] | X = 1.003, df = 5.663, p = 0.9798 |
| 65-69 | ARIMA(1,1,1)(2,0,1)[12] | X = 3.9818, df = 5.663, p = 0.6388 |
| 70-74 | ARIMA(3,1,1)(2,0,2)[12] | X = 1.0118, df = 5.663, p = 0.9794 |
| 75-79 | ARIMA(3,1,3)(1,0,1)[12] | X = 0.1189, df = 5.663, p = 1 |
| 80-84 | ARIMA(2,1,1)(2,0,2)[12] | X = 4.2804, df = 5.663, p = 0.5973 |
| 85+ | ARIMA(1,1,2)(2,0,0)[12] | X = 0.3565, df = 5.663, p = 0.9986 |
| Ischemic heart disease |  |  |
| 0-4 | ARIMA(0,1,1)(1,0,0)[12] | X = 2.3994, df = 5.663, p = 0.854 |
| 5-54 | ARIMA(1,1,2)(2,0,1)[12] | X = 1.236, df = 5.663, p = 0.9665 |
| 55-64 | ARIMA(2,1,3)(1,0,2)[12] | X = 1.5871, df = 5.663, p = 0.94 |
| 65-69 | ARIMA(4,1,3)(1,0,1)[12] | X = 1.4818, df = 5.663, p = 0.9487 |
| 70-74 | ARIMA(2,1,1)(2,0,2)[12] | X = 0.9129, df = 5.663, p = 0.984 |
| 75-79 | ARIMA(2,1,1)(1,0,2)[12] | X = 5.2412, df = 5.663, p = 0.4713 |
| 80-84 | ARIMA(1,1,0)(1,0,1)[12] | X = 35.1582, df = 5.663, p<0.001 |
| 85+ | ARIMA(2,0,4)(0,0,1)[12] | X = 1.343, df = 5.663, p = 0.9592 |
