# Supplementary material for: Excess Mortality Associated with Influenza Epidemics in Portugal, 1980 to 2004
Source: PLoS One. 2011 Jun 21;6(6):e20661. doi: 10.1371/journal.pone.0020661 (PMC3119666; doi:10.1371/journal.pone.0020661)
Supplement: Table S4 — Correlation matrix between seasonal age-standardized excess rates. Injuries are used as a control time series which should not be associated with influenza virus circulation. (DOCX) [file pone.0020661.s014.docx]

Table S4 - Correlation matrix between seasonal age-standardized excess rates. Injuries are used as a control time series which should not be associated with influenza virus circulation.

|  | All causes | CVD | IHD | DRS | PI | CRD | ILI(1) | **Injuries** |
| --- | --- | --- | --- | --- | --- | --- | --- | --- |
| All causes | 1 | 0.950* | 0.845* | 0.926* | 0.950* | 0.824* | 0.765* | **0.026** |
| CVD |  | 1 | 0.783** | 0.786* | 0.857* | 0.652* | 0.641* | **-0.082** |
| IHD |  |  | 1 | 0.855* | 0.828* | 0.806* | 0.829* | **0.094** |
| DRS |  |  |  | 1 | 0.952* | 0.949* | 0.807* | **0.079** |
| PI |  |  |  |  | 1 | 0.838* | 0.743* | **-0.0002** |
| CRD |  |  |  |  |  | 1 | 0.794* | **0.151** |
| ILI(1) |  |  |  |  |  |  | 1 | **0.426** |
| Injuries |  |  |  |  |  |  |  | **1** |

CVD: cardiovascular disease; IHD: ischemic heart disease; DRS: diseases of the respiratory system: PI: Pneumonia and Influenza; CRD chronic respiratory disease: * p<0.05; (1) correlation with ILI was only performed for the group of 65 and plus years of age
