# Supplementary material for: Excess Mortality Associated with Influenza Epidemics in Portugal, 1980 to 2004
Source: PLoS One. 2011 Jun 21;6(6):e20661. doi: 10.1371/journal.pone.0020661 (PMC3119666; doi:10.1371/journal.pone.0020661)
Supplement: Table S5 — Sensitivity analysis: excess deaths and age-standardized excess death rates from injuries that are “attributable to influenza” by our method. Injuries comprise all external causes of death. (DOCX) [file pone.0020661.s015.docx]

Table S5 –Sensitivity analysis: excess deaths and age-standardized excess death rates from injuries that are “attributable to influenza” by our method. Injuries comprise all external causes of death.

| Season | Excess (absolute number) | Age-standardized excess rate (/10^5) | Proportion of excess (oct-may) | All external | All external rate |
| --- | --- | --- | --- | --- | --- |
| 1980/1981 | 47 | 0.40 | 1.0% | 4547 | 42.1 |
| 1981/1982 | 30 | 0.20 | 0.5% | 4783 | 44.2 |
| 1982/1983 | 15 | 0.12 | 0.3% | 4650 | 42.0 |
| 1983/1984 | 29 | 0.15 | 0.4% | 4628 | 41.6 |
| 1984/1985 | 0 | 0.00 | 0.0% | 4472 | 39.7 |
| 1985/1986 | 16 | 0.08 | 0.2% | 4265 | 37.4 |
| 1986/1987 | 15 | 0.07 | 0.2% | 4461 | 39.4 |
| 1987/1988 | 0 | 0.00 | 0.0% | 4420 | 42.1 |
| 1988/1989 | 15 | 0.09 | 0.2% | 4393 | 44.2 |
| 1989/1990 | 32 | 0.23 | 0.7% | 4097 | 42.0 |
| 1990/1991 | 30 | 0.31 | 0.8% | 4255 | 41.6 |
| 1991/1992 | 0 | 0.00 | 0.0% | 4338 | 39.7 |
| 1992/1993 | 0 | 0.00 | 0.0% | 4059 | 37.4 |
| 1993/1994 | 0 | 0.00 | 0.0% | 3672 | 39.4 |
| 1994/1995 | 0 | 0.00 | 0.0% | 3654 | 42.1 |
| 1995/1996 | 30 | 0.16 | 0.5% | 3655 | 44.2 |
| 1996/1997 | 18 | 0.12 | 0.4% | 3590 | 42.0 |
| 1997/1998 | 18 | 0.11 | 0.4% | 3509 | 41.6 |
| 1998/1999 | 37 | 0.14 | 0.5% | 3318 | 39.7 |
| 1999/2000 | 27 | 0.19 | 0.8% | 3199 | 37.4 |
| 2000/2001 | 0 | 0.00 | 0.0% | 3055 | 39.4 |
| 2001/2002 | 277 | 2.56 | 8.4% | 3865 | 42.1 |
| 2002/2003 | 26 | 0.17 | 0.6% | 3684 | 44.2 |
| 2003/2004 | 0 | 0.00 | 0.0% | 3518 | 42.0 |
| Mean | 28 | 0.21 | 0.66% | 4004 | 41.2 |
